# Alternative way to test the efficacy of swine FMD vaccines: measurement of pigs median infected dose (PID_50_) and regulation of live virus challenge dose

**DOI:** 10.1186/1743-422X-7-215

**Published:** 2010-09-08

**Authors:** Dong Li, Zeng-Jun Lu, Bao-Xia Xie, Pu Sun, Ying-Li Chen, Yuan-Fang Fu, Zai-Xin Liu

**Affiliations:** 1 Key laboratory of Animal Virology of Ministry of Agriculture, National Foot-and-Mouth Disease Reference Laboratory of China, State Key Laboratory of Veterinary Etiologic Biology, Lanzhou Veterinary Research Institute, Chinese Academy of Agricultural Sciences, Lanzhou, Gansu, 730046 China

## Abstract

Foot-and -mouth disease to pigs is serious recently around the world. "Vaccination prevention" is still an important policy. OIE specifies 10,000 TCID_50_(0.2 ml) of virulent virus for challenge test in pigs to test the potency of FMD vaccine by intradermal route inoculating the virus in the heel bulbs of one foot or by intramuscular route administering into one site of the neck behind the ear. Convenience and speediness are available in the process of potency test of commercial FMD vaccine. We selected the route of "administering into one site of the muscular part of the neck behind the ear" because of convenience and speediness. However, it was difficult to infect control pigs even up to 100,000TCID_50_, so we changed the challenged virus from cell-passaged strain to suckling mice-passaged one, measured its PID_50 _(pigs median infected dose) and defined the virus challenge dose as 1000PID_50_. Meanwhile, we arranged the number of control pigs from two to three for easy evaluation.

## Findings

Foot-and-mouth disease (FMD) is an extremely contagious viral disease of cloven-hoofed domesticated as well as wild animals and has a great potential for causing severe economic loss. The causal agent, FMD virus (FMDV), is a member of the genus *Aphthovirus *in the family *Picornaviridae *and occurs as seven distinct serotypes throughout the world: A, O, C, Asia1 and South African Territories (SAT) 1-3. Vaccination is the most important control and eradication strategy for FMD, especially the oil-adjuvant vaccine in developing countries [1-4]. The different processes for preparing vaccines against viral diseases are comprised by a sequence of steps which, although different in accordance with particular virus and processes selected, may be classified as follows: virus production, virus inactivation and vaccine formulation.

In recent years, the pigs infected with FMD have been reported around the Chinese mainland, such as Chinese Taipei, Chinese Hong Kong [5-9]. Furthermore, import of vaccines are not only expensive, they are not always suitable. Therefore research into vaccine development and improvement is vital. In order to test vaccine efficacy in swine, the protected rate as well as ID_50 _and the challenge dose need to be determined. The OIE Manual of Diagnostic Tests and Vaccines for Terrestrial Animals (OIE terrestrial manual 2009, Chapter 2.1.5 Foot-and-mouth disease) lists a number of criteria for testing FMD vaccines in pigs. These include challenge in the heel bulb of 1 foot with 10^4 ^TCID_50 _of FMDV titered in a suitable pig cell culture systems or administering into one site of the muscular part of the neck behind the ear. However, "challenge in the heel bulb" is too laborious and time-consuming during the commercial vaccine potency tests because more than hundred pigs need to be captured at the same time, so we choose "administering into one site of the muscular part of the neck behind the ear" as our challenge route. Most of the FMD vaccine companies in China select BHK-21 cell lines to propagate the swine vaccine virus strain, and we also use BHK-21 passaged virus as the challenge strain to keep the consistency. Unfortunately, we did not achieve a good result in our factual operation due to the control pigs not appear clinical symptoms while challenged intramuscularly with 10000 TCID_50_(TCID_50 _= 8.0), which means that the tests did not go on. Therefore we tried to change the virus from cell-passaged strain to suckling mice-passaged strain and hope to get a good result.

FMDV OH/99 strain was isolated from pigs [10] and propagated in suckling mice as the challenge strain and in baby hamster kidney (BHK-21) cells as the vaccine strain. The suckling mice of 2-3-day-old were subcutaneously injected with 0.1 ml of OH/99 strain and passed for 3-5 passages. Upon death (16-20 hr post-infection), the bodies were collected and grinded.

Pigs median infected dose (PID_50_) was determined with suckling mice's passaged strain OH/99. The strain was series decuple diluted from 10^-1 ^to 10^-10^. Forty 2-month-old pigs, sero-negative for FMDV antibodies, were randomly divided into ten groups. Each group including four pigs was housed in a separate room and inoculated at the ear-root-neck area with 2 ml virus-dilution per pig from 10^-1 ^to 10^-10 ^respectively. After ten days clinical observation, the PID_50 _was calculated according to Karber method. The experiment was repeated for the dilutions 10^-5 ^to 10^-8^. The PID_50 _was calculated to be 6.5. The data were shown in Table 1. The repeated data were the same.

**Table 1:** Data for calculating PID_50_

Virus dilution	Number of pigs	Number of healthy pigs	Number of sick pigs
10^-1^	4	0	4
10^-2^	4	0	4
10^-3^	4	0	4
10^-4^	4	0	4
10^-5^	4	0	4
10^-6^	4	1	3
10^-7^	4	3	1
10^-8^	4	4	0
10^-9^	4	4	0
10^-10^	4	4	0

The virus culture liquids were treated by ethylenimmine 0.035 M/L at 30°C during 24 h for virus inactivation, then the inactivation was stopped by 0.04 M sodium thiosulphate [11]. Following the operation guidelines, the inactivated FMDV antigen was emulsified with Montanide ISA 206(Seppic, France)oil.

Sixteen 2-month-old pigs, sero-negative for FMDV, were intramuscular inoculated at the ear-root-neck area with 2 ml inactivated vaccine respectively, and three pigs were bred without vaccination in the same room as negative control. Sera samples were collected at 14 dpv (days post vaccination) and 28 dpv to assay the antibody against FMDV serotype O using a standard LPB-ELISA (Liquid phase blocking-ELISA).

To demonstrate vaccine efficacy, all 19 pigs were challenged intramuscularly with 1000 PID_50_/2 ml of FMDV OH/99 suckling mice passaged strain at the ear-root-neck area after 28 dpv and FMD symptoms were monitored for 10 days.

The results of the antibody response titrations of 14 dpv and 28 dpv of vaccinated pigs by LPB-ELISA and protective effect in pigs were recorded in table 2. The average titer at 14 dpv and 28 dpv was 1.50 and 1.84 respectively. At 2-4 days after challenge, blisters were observed on three animals of the negative control group. Sixteen vaccinated pigs were completely protected.

**Table 2:** The results of the sera titers of 14 dpv and 28 dpv of vaccinated pigs by LPB-ELISA and protective effect in swine challenged with OH/99

No. of pig	1	.2	3	4	5	6	7	8	9	10	11	12	13	14	15	16	17 (Control)	18 (Control)	19 (Control)
Titer 14 dpv(-log10)	1.34	1.34	1.04	1.34	1.81	1.65	1.04	1.04	1.04	1.04	1.65	1.34	1.34	1.65	1.65	1.65	< 0.6	< 0.6	< 0.6
Titer 28 dpv(-log10)	1.81	1.95	1.65	1.81	2.25	2.11	1.65	1.34	1.65	2.11	1.95	1.81	1.95	1.81	2.11	1.81	< 0.6	< 0.6	< 0.6
Challenge	P**^a^**	P	P	P	P	P	P	P	P	P	P	P	P	P	P	P	U**^b^**	U	U

^a ^P means the pig was protected after challenge 28 dpv

^b ^U means the pig was unprotected after challenge 28 dpv

There are two prevention and eradication strategies for FMD. "Slaughter policy" is definitely widely used in the developed countries, but "vaccination policy" is carried out in developing countries due to high FMD prevalence and economic reasons. The FMD, in most of the spontaneous epidemic areas, was found to spread from cattle to cattle. However, in recent years, pigs' FMD circulated. China is one of the largest swine breeding countries and pork plays a very important role in people's daily life. In view of the above mentioned arguments, the development of a cheap FMD vaccine for swine is warranted and urgently needed. After all, swine and cattle are different species, and they have different immune system. The antibody response level against FMDV type O in swine is lower and weaker than which in cattle [12]. Normally, 10000 ID_50 _of virus challenge dose was used in cattle FMD vaccine efficacy testing and 10000TCID_50 _was used in pigs FMD vaccine efficacy testing (OIE terrestrial manual 2009, Chapter 2.1.5 Foot-and-mouth disease). However, in our experiments, 10000 ID_50 _dose was too strong in pigs as even high efficient vaccines did not protect the animals but 10000TCID_50 _was too weak in pigs even 10^5 ^TCID_50 _only cause part of control pigs occurring clinical signs, which was difficult to judge the result's credibility while "administering into one site of the muscular part of the neck behind the ear "(interior data). The pig's original FMD virus was stronger and could be used as the challenge strain, but a large amount of the materials was limited to obtain. The suckling mice passaged virus was used here as challenge strain to solve the problem. Comparatively, the suckling mice passaged virus was easier to propagate, and the virulence equal to original virus but stronger than BHK-21 passaged one, and infected all control pigs in our repeatedly experiments. Therefore, the suckling mice passaged virus was applied in our intramuscular FMD vaccine potency test and a challenge dose was considered to be 1000 PID_50_.

Two of control pigs were arranged in OIE terrestrial manual 2009. We found that sometimes one pig occurred FMD clinical symptoms but another not while challenge even with original virus. The reason was not clear. But how to evaluate the potency test when 1/2 of control is positive? So we set three pigs as control. The test will be appropriate when 2/3 of control pigs generated disease.

The antibody titer is one of referenced criteria to evaluate vaccine potency as it is positively linked with protection rate, but it is influenced by factors such as vaccine's antigen content, animal's individual status, virus challenge dose and route [13-15]. The LPB-ELISA assay was recognized as one of the standard methods to detect antibody. The kit (bought from WRLforFMD, Britain) was handled according to manufacture's instruction. When the titer is higher than 1.65, it means positive. Lower than 0.6 means negative. The range from 0.78 to 1.65 is a gray area, indicated that some animals can be protected, others can not. In our result, only one pig's (No.8) antibody titer (1.34) of 28 dpv was lower than 1.65, which belong to higher titer of gray area, and it was protected in the challenge. Although these data criteria were designated to cattle, we referred it to pigs here.

However, the antibody titers could not instead of PD_50 _test. There are two ways to test FMD vaccine potency: PD_50 _test(within Europe) or PG test (Protection against Generalization [16]. Here, we accepted the ratio of vaccine protection which used widely in South America. Sixteen are vaccinated for 28 days, and other three are negative control. The vaccine final products will be qualified when the protected rate is equal or more than 12/16, and sickness rate for control is equal or more than 2/3. Here, the vaccine's protection rate was 16/16.

From above, we measured pigs median infected dose (PID_50_), used suckling mice passaged virus as intramuscular challenged strain, and determined 1000 PID_50 _as the challenge dose to test the efficacy of swine FMD vaccine.

## Competing interests

The authors declare that they have no competing interests.

## Authors' contributions

ZXL is the leader of the study group. DL carried out the experiments and wrote the manuscript. ZJL and PS carried out the animal tests. BXX and YLC propagated the FMDV strain. YFF detected all the sera titers. All authors read and approved the final manuscript.
